# Feasibility of an automated telephone survey to enable prospective monitoring of subjects whose confidentiality is paramount: a four-week cohort study of partner violence recurrence after Emergency Department discharge

**DOI:** 10.1186/1742-5573-5-1

**Published:** 2008-01-07

**Authors:** Douglas J Wiebe, Brendan G Carr, Elizabeth M Datner, Michael R Elliott, Therese S Richmond

**Affiliations:** 1 Department of Biostatistics and Epidemiology, University of Pennsylvania School of Medicine, Philadelphia, PA, USA; 2 The Robert Wood Johnson Clinical Scholars Program, Department of Emergency Medicine, University of Pennsylvania School of Medicine, Philadelphia, PA, USA; 3 Department of Emergency Medicine, University of Pennsylvania School of Medicine, Philadelphia, PA, USA; 4 Department of Biostatistics, University of Michigan School of Public Health, Ann Arbor, MI, USA; 5 School of Nursing, University of Pennsylvania, Philadelphia, PA, USA

## Abstract

**Objective:**

A goal in intimate partner violence (IPV) research is to identify victims when they are treated in a hospital Emergency Department (ED) and predict which patients will sustain abuse again after discharge, so interventions can be targeted. Following patients to determine those prognostic factors is difficult, however, especially to study IPV given the risk to be assaulted if their partner learns of their participation. We assessed the feasibility of an automated telephone survey and a wireless incentive delivery system to follow ED patients after discharge, enabling detection of IPV recurrence.

**Methods:**

A four-week prospective cohort pilot study was conducted at an urban academic medical center ED in the U.S. Thirty patient subjects (24 women, 6 men; 18–54 years) who had sustained IPV in the past six months, 12 of whom presented for an acute IPV-related condition, were interviewed in the ED and were asked to report weekly for four weeks after discharge to a toll-free, password protected telephone survey, and answer recorded questions using the telephone keypad. A $10 convenience store debit card was provided as an incentive, and was electronically recharged with $10 for each weekly report, with a $20 bonus for making all four reports.

**Results:**

Twenty-two of 30 subjects (73.3%) made at least one report to the telephone survey during the four weeks following discharge; 14 of the 30 subjects (46.7%) made all four weekly reports. Each time the telephone survey was accessed, the subject completed all questions (i.e., no mid-survey break-offs). Eight months after follow-up ended, almost all debit cards (86.7%) had been used to make purchases.

**Conclusion:**

Approximately three of every four subjects participated in follow-up after ED discharge, and approximately two of every four subjects completed all follow-up reports, suggesting the method of an automated telephone survey and wireless incentive delivery system makes it feasible to study IPV prospectively among discharged patients. That finding, along with evidence that IPV recurrence risk is high, suggests the protocol tested is warranted for use conducting full-scale studies of IPV. The protocol could benefit efforts to study other outcomes, especially when patient confidentiality is paramount for their safety.

## Introduction

Follow-up of patients who visit the Emergency Department (ED) or other healthcare site is difficult, especially to monitor recurrence of intimate partner violence (IPV). In addition to facing logistical challenges germane to follow-up research in general, follow-up research involving participants known to be in an abusive relationship raises an additional, paramount concern: the participant's partner may be angered and may respond with abuse if he or she becomes aware of the fact that the participant is involved in a study of IPV.

The interest in IPV recurrence stems from the reality that many victims of violence do not come to the attention of the healthcare system until they are treated in a hospital ED [1], and this contact creates an opportunity for researchers and clinicians to help prevent further abuse once the patient leaves the hospital. Thus the interest in follow-up methodology, both to identify prognostic factors for recurrence and to test interventions for secondary prevention.

The efficacy of intervention efforts launched in the ED setting with the goal of preventing IPV recurrence is largely unknown [2], however, owing to the logistical and ethical challenges of following up with patients at risk for IPV [3]. Moreover, these same challenges have hampered efforts simply to obtain valid estimates of the incidence of IPV recurrence after hospital discharge and to identify factors that predict IPV recurrence [4,5]. In the past, IPV recurrence has been studied by identifying those ED patients who made a second visit to the ED [4]. A shortcoming of such an approach is that visits to non-participating hospitals, or to a hospital with a registry that is not coordinated with the hospital of discharge, will not be detected, nor will instances of IPV that do not prompt an ED visit. Other studies have determined IPV recurrence by having members of the study team initiate telephone calls to patients after discharge [5]. This approach carries the risk of exposing the patient's involvement in IPV research to the abusive partner, thus putting the patient at risk to be harmed. One option is to initiate the phone contact only after substantial time has passed (i.e., months or years) [5], perhaps based on the expectation that recurrences will be rare and thus require a long follow-up period to study with statistical tests, or that safety will be conferred by delaying the contact with the patient until long after the ED visit took place. This could cause imprecision and recall bias, however, if the lag limited the patient's recollection of the timing and nature of IPV recurrences.

If a mechanism were available to obtain valid information about the health and wellbeing of patients after discharge in a manner that does not compromise patient safety, this would create opportunities for epidemiologic and intervention studies in any number injury research areas. In the context of IPV, the studies that would be enabled by such a mechanism could provide valuable guidance for how ED protocols could best manage patient victims of IPV with the goal of minimizing risks for IPV recurrence [6].

The objective of this pilot study was to assess whether an automated telephone survey and a wireless incentive delivery system can be used by investigators as a means that makes it possible to prospectively monitor the recurrence of IPV among ED patients during the weeks following hospital discharge.

## Methods

Participants were recruited from the ED of the Hospital of the University of Pennsylvania, an urban academic medical center in the U.S., during a three-month period of 2006. Screening for eligibility was conducted by the Academic Associates program, which consists of a cadre of trained and supervised students who staff the ED daily between the hours of 0700 and midnight solely for the purpose of screening [7,8]. Criteria for inclusion were age (18–64 years old) and presenting to the ED for an IPV-related injury or illness, or having sustained IPV within the past six months. IPV was assessed using a four-item questionnaire adapted from the Abuse Assessment Scale [9,10], and was defined as coercion, intimidation, or injury inflicted verbally, physically or sexually by a current or former boyfriend, girlfriend, husband or wife (same sex or opposite sex).

A total of 46 patients met eligibility criteria. Sixteen patients did not wish to participate. The remaining 30 patients (65.2%) participated as study subjects and completed the baseline interview. Characteristics of the subjects are shown in Table 1.

**Table 1:** Baseline characteristics of study participants (N = 30)

Characteristic		No. of subjects
Age in years, mean (SD)		30 (11)

Sex		
	Women	24 (80.0%)
	Men	6 (20.0%)

Race		
	African America	26 (87.0%)
	Caucasian	3 (10.0%)
	Other	1 (3.3%)

Chief complaint at triage		
	Abdominal pain	4 (13.3%)
	Abscess	2 (6.7%)
	Assault	4 (13.3%)
	Asthma	1 (3.3%)
	Back pain/migraine	1 (3.3%)
	Carbon monoxide poisoning	1 (3.3%)
	Coughing up blood	1 (3.3%)
	Dizzy	2 (6.7%)
	Hip pain	1 (3.3%)
	Jaw pain (broken)	1 (3.3%)
	Laceration	1 (3.3%)
	Nauseous/vomiting	2 (6.7%)
	Pregnant and cramping	3 (10.0%)
	Rectal bleeding	1 (3.3%)
	Seizure	1 (3.3%)
	Stab wound	1 (3.3%)

Presenting for IPV		11 (36.7%)

Partner's relationship patient subject		
	Boyfriend/girlfriend	14 (46.7%)
	Spouse	3 (10.0%)
	Ex-boyfriend/ex-girlfriend/ex-spouse	8 (26.7%)
	Child's father/mother	5 (16.7%)

IPV frequency during past six months		
	Daily	5 (16.7%)
	Multiple times per week	7 (23.3%)
	Less frequently	18 (60.0%)

Most recent instance of IPV (harm or threaten) with weapon^†^		
	Today	3 (10.0%)
	This week	4 (13.3%)
	Last two weeks	1 (3.3%)
	This month	2 (6.7%)
	Past six months	7 (23.3%)
	Never	12 (40.0%)
	Don't know	1 (3.3%)

Most recent instance of being injured (IVP) with a weapon^‡^		
	Today	2 (6.7%)
	This week	2 (6.7%)
	Last two weeks	1 (3.3%)
	This month	2 (6.7%)
	Past six months	9 (30.0%)
	Never	13 (43.3%)
	Don't know	1 (3.3%)

BDI-FastScreen score, mean (SD)		9.6 (5.9)

Note: BDI denotes Beck Depression Inventory. The suggested guidelines for scoring, when the mean score is approximately 8 and the standard deviation is approximately 4, are 0–3 (minimal), 4–8 (mild), 9–12 (moderate), 12–21 (severe). [20]

^† ^Responses were: 2 × 4 board, bat, beer bottle, belt, car, gun, knife, plate, television remote control, shoe, stick, wallet.

^‡ ^Responses were: ash tray, bat, belt, broomstick, fist, gun, knife, plate, stick, television remote control, shoe, dresser, scalpel, wallet.

Enrollment, consent, and interviewing of the 30 subjects was conducted by one investigator (DW), who was paged and responded in person to the ED each time an eligible patient was identified. The interview was conducted in the ED in private and involved administering an 18-item questionnaire which solicited information on the frequency and nature of IPV that the subject had sustained in the past six months, and included a brief screener for depression (BDI-FastScreen[11]) following from our team's interest in mental health.

As part of the interview, it was explained to subjects that our study team was concerned about the health and safety of patients after they leave the ED, and that we were testing an automated telephone survey and a wireless incentive delivery system to assess whether it would provide an effective and safe way to obtain information from patients after they are discharged from the ED. Subjects were asked if, over the four weeks following discharge, they would report weekly to the automated telephone survey being tested. Subjects were given a business-card sized information card that listed the telephone number to call to connect to the survey, and was generic (i.e., gave no indication of being linked to a research study). Subjects were asked to designate a four-digit number to be used as their password to gain access, which the investigator later programmed into the survey system. Calendars for the current month and subsequent month were printed on the back of the card. The investigator circled the dates one, two, three, and four weeks into the future as a reminder of when to call in to the automated telephone survey, and asked the subject to report on those dates, or as close to those dates as was convenient, at any time of day or night as was convenient. Subjects were also given a copy of the investigator's business card and were instructed to call his cellular phone any time in the event of losing the information card or having questions about the study.

At the conclusion of the interview, each subject was asked if it would be okay for the investigator to call the subject at a phone number of their choice if it was determined that the subject had not made a report to the system for two weeks. The purpose of including this question in the interview was to assess each subject's willingness to receive a telephone call for research purposes, which could be used as was a way to maximize retention of the sample. It was described to subjects that, at that time, the investigator could remind the subject of the telephone number to call in case the information card that had been provided had been lost. At this point in the conversation the investigator reiterated being concerned with the subject's safety and the potential that a call to the subject could reveal to the subject's partner their participation as a research participant.

A debit card redeemable at a convenience store chain was given as an incentive to subjects at the conclusion of the baseline interview. The debit card contained $10 in credit. After ED discharge, a disbursement of $10 was made electronically to a subject's debit card shortly after each report was made to the telephone survey (within one or two days); subjects who made all four weekly reports were disbursed an additional $20 in credit as a bonus.

Each subject also received a pamphlet of information, including telephone numbers and resources, for victims of IPV. This was given to the patient by medical staff as part of standard clinical practice, however, rather than as part of the research protocol. The study was approved after full review by the Institutional Review Board of the University of Pennsylvania.

### Automated telephone survey

The follow-up survey was hosted by a survey research firm and was administered via a toll-free number using an automated, Interactive Voice Response (IVR) telephone survey consisting of recorded questions that could be answered YES or NO by pressing buttons on the telephone keypad. The survey script recording was performed by an adult female and opened with a generic welcome message and a prompt for a password. The questions asked whether, during the time since discharge or since their last report to the telephone survey, the subject had sustained a new IPV incident and whether the subject had sought medical care for a condition related to a new IPV incident. The question wording is shown in Table 2. The data reported to the survey were accessed by the research team via a password-protected website hosted by the survey research firm.

**Table 2:** Intimate partner violence questions administered during follow-up via automated telephone survey

• Afraid of intimate partner
In the week since you [were seen in the hospital/made your last call to this survey], have you been afraid of your partner?
• Verbal IPV
People can use words in ways that hurt. In the week since you [were seen in the hospital/made your last call to this survey], has your partner used WORDS to harm you, to threaten you, or to coerce you to do something you didn't want to do?
• Physical IPV
In the week since you [were seen in the hospital/made your last call to this survey], has your partner pushed you or hit you with their hands, fists or feet?
• Weapon-related IPV
People can use many types of objects as weapons, including knives and guns and also many household objects. In the week since you were seen in the hospital/made your last phone call to this survey, has your partner used something as a weapon to harm you, to threaten you, or to coerce you to do something you didn't want to do?
• Medical care seeking
In the week since you [were seen in the hospital/made your last call to this survey], did you seek medical care for injuries or for illnesses or stress caused by threats or your relationship?

IPV denotes intimate partner violence.

Participants responded by pressing 1 for YES and 2 for NO.

### Outcome measures

The primary outcomes were whether and how often subjects reported to the telephone survey weekly after discharge. Also of interest was the time of day and day of week that reports to the telephone survey were made, and whether subjects made use of the debit card incentive to make purchases; this was monitored via an online system hosted on the convenience store's website. Secondary outcomes corresponded to the risks of sustaining IPV or seeking medical care during follow-up, as ascertained by the questions administered via the automated telephone survey.

### Subject safety

Considerable planning went into developing the incentive delivery method and other aspects of the research protocol such that all potential risks that were identified were minimized. Strategies in addition to those referred to above (e.g., making the information card generic) included:

- During the informed consent process, statements were made to ensure that the patient was aware that keeping a copy of the consent form could put the patient at risk if found by their partner, and that the patient was not required to keep a copy of the consent form.

- Instead of administering a separate HIPAA (Health Insurance Portability and Accountability Act, a federal law regulating the use of health information) form as the patient was being recruited, the elements of HIPAA were incorporated into the informed consent form administered in the ED. In addition to streamlining the recruitment process, this was done to avoid creating an additional form that revealed that a study was being conducted on IPV, and that the patient would be given the opportunity to keep, which could lead to the patient's involvement in the study coming to the attention of the patient's intimate partner.

- Anyone calling the toll-free telephone number of the IVR survey heard only a generic recorded statement and a prompt to enter a password (akin to "Welcome to the Patient Line at Penn. Please enter your password."). This prevented non-study participants from accessing the telephone survey, and prevented non-study participants from learning that the purpose of the telephone number was to provide access to a survey on IPV.

- Subjects accessed the IVR survey by entering at the prompt a password that was agreed upon with the interviewer at the time of the interview conducted in the ED. This was a four digit, numeric password that the patient was asked to specify. During the interview, the interviewer cautioned the patient to not write down their password in order to avoid the potential for their intimate partner to gain access to the survey.

- Anyone who called the toll-free telephone number for the IVR survey and who entered an incorrect password at the prompt, or did not enter a password, heard a recorded message providing a telephone number to call for information about the "Patient Line." Anyone who called that number heard a recorded message asking the caller to leave their first name, a telephone number where they could be reached, and times to call. This was the number of the investigator's cellular telephone. The investigator checked this voicemail account daily, calling back patients if a call was missed, and reminding them of their password to access the survey. Before providing a password to any individual, a series of short questions was asked to crosscheck against information collected in the ED to confirm the caller was who they claimed to be.

- When a subject accessed the IVR survey by entering the correct password, she heard a statement to remind her that she could hang up to end the call at any time, without any negative consequences, if she felt unsafe.

- The convenience store chain that redeemed the debit cards used as incentives sells food, beverages, merchandise and gasoline, but does not sell alcohol, which is important in this context given that alcohol consumption is a risk factor for IPV.

- The convenience store chain had not previously hosted a method to recharge debit cards, as was done during the follow-up portion of the study. During the planning phase of the study, the investigator (DW) approached executives at the store chain, described the study design and the importance of devising a safe way to deliver incentives to subjects, and requested that the recharge capability be developed and made available to the investigator through an on-line interface. The executives graciously arranged for this.

- In the event a debit card incentive was lost, by calling the investigator and confirming his identity according to questions asked during the in-person interview conducted in the ED, the investigator and subject could discuss whether it was safe for the investigator to mail a replacement debit card to the subject at an address of the subject's choosing.

### Statistical analysis

Participation was calculated as the proportion of subjects who reported at least one time to the telephone survey during the four weeks following discharge, the proportion of subjects who made all four weekly reports to the telephone survey, and the proportion of reports to the telephone survey that were complete (i.e., not terminated early). Percentages were used to evaluate whether reporting varied by time of day or day of week. Data resulting from the reports to the telephone survey were analyzed using the product limit method and plotted (i.e., Kaplan-Meier curves) [12] to estimate the event-free proportion (five events: not being afraid of intimate partner; not sustaining verbal IPV; not sustaining physical IPV; not sustaining IPV using a weapon; not seeking medical care for IPV-related harm) over the four-week follow-up period and to estimate the cumulative risk of IPV or seeking medical care (i.e., 1 minus event-free proportion)[13] at one week and four weeks post discharge. The survival and risk estimates were computed with end points defined as the first recurrence of each outcome. Subjects were censored at 28 days after ED discharge if they made a report to the telephone survey after that time; subjects also were censored if they last reported to the survey within 28 days of discharge, in which case they were censored on the day of the last report. To enable the calculations, reports of IPV were assumed to have occurred on the date halfway between the date of the report and the date of ED discharge or the previous report.

## Results

Twenty-two (17 women; 5 men) of the 30 subjects (73.3%) made at least one report to the automated telephone survey during the four weeks following ED discharge. Among the 22 subjects who made at least one report, 14 subjects (63.6%) made all four weekly reports. Among the 30 subjects overall, the percent of subjects making 0, 1, 2, 3, or 4 reports to the telephone survey was 26.7%, 20.0%, 3.3%, 3.3%, and 46.7%, respectively. Each time the telephone survey was accessed, the subject completed all questions (i.e., no mid-survey break-offs).

There was contact between the research team and eight of the 30 subjects during the follow-up period. Five subjects contacted the investigator (DW) by calling his cellular phone to be reminded of either the telephone number for the automated survey or for their password to gain access. The other three instances involved the investigator making a telephone call to subjects who had not made a report to the automated telephone survey for two weeks. The decision to initiate this contact was made only after careful consideration of information that subjects provided during the interview in the ED. While in the ED, all 30 subjects responded that they were willing to be called by the investigator. However, two of the subjects had neither a home telephone nor a cellular telephone. Many others stated stipulations for when to call and which number to call. Two subjects reported that they currently lived with an abusive partner, but that it was okay to call the home telephone because the subject usually answered the phone. In total, 15 subjects listed a home telephone as the phone to call, and in two instances this was the phone at their parents' homes, where the subject was staying for respite from their partner. Ten subjects listed a cellular telephone as the phone to call, but one of these subjects reported that the number may be inactive because she changed her cellular phone number regularly to avoid contact with her partner. Three subjects listed a work telephone as the phone to call. Overall, 10 of the 28 subjects providing telephone numbers indicated specific times to call or to not call, with the remaining subjects saying it was okay to call any time.

Of the three subjects called by the investigator, two were reached immediately. Both expressed being willing to report to the phone survey and indicated simply forgetting to do so, and were found to have reported shortly afterward. The other subject could not be reached, and was said by an individual who answered the phone to have moved. Regarding the two subjects who during the interview in the ED indicated having no home phone or cellular phone, neither made reports to the automated telephone survey.

**Figure 1:**
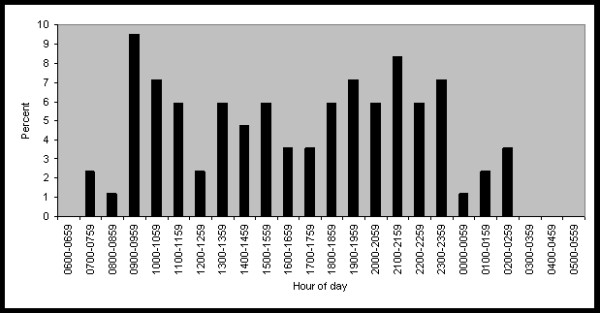
Percent of subjects' reports to automated telephone survey during follow-up, by hour of day.

Information on the time of day when reports were made to the telephone survey is shown in Figure 1. More reports (9.5%) were made during the morning hour of 0900–0959 than during any other hour of the day, and a large proportion of calls were also made in the evening, with 8.3% of reports made during the 2100–2159 hour. No reports were made between the early morning times of 0300 and 0659. In general it was more common for reports to be made on weekdays than on weekends. The proportion of reports made by day of week are as follows: Sunday 9.4%, Monday 15.3%, Tuesday 22.4%, Wednesday 16.5%, Thursday 15.3%, Friday 14.1%, Saturday 7.1%.

When reviewed approximately eight months after conclusion of the follow-up period, almost all of the debit cards provided as incentives (26 of 30; 86.7%) had been used to make convenience store purchases. Of those debit cards that had been used, 13 had been exhausted ($0.00 remaining); the greatest dollar amount still available was approximately $5 and the average dollar amount still available was minimal ($0.69).

**Figure 2:**
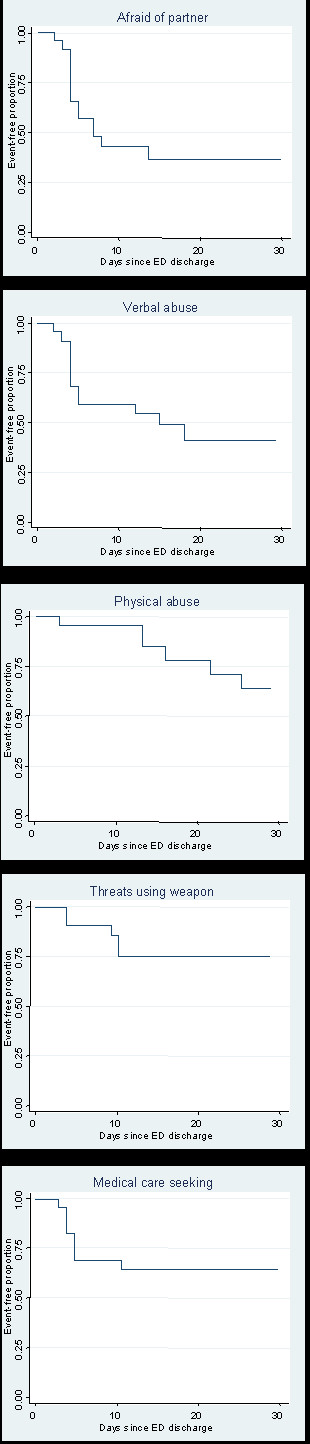
Kaplan-Meier estimates of survival during four weeks following ED discharge.

The four-week survival experience of the subjects, which indicates the proportion not experiencing the outcomes of interest after ED discharge, is shown in Figure 2. The graphs reveal that, for example, it was common for subjects to have been afraid of their partner within the first two weeks of discharge, after which time few additional subjects reported a first instance of being afraid. Also, the risk of physical abuse generally became more common over the 4-week follow-up period. Estimates of the cumulative risks of each outcome at one-week and four-weeks following discharge are shown in Table 3. Regarding the question about weapon-related IPV, six of the 22 subjects who reported to the telephone survey indicated experiencing weapon-related IPV during the follow-up period, and one of those six subjects indicated upon at least one report that the weapon was a firearm.

**Table 3:** Cumulative risk of IPV and seeking medical care during four weeks following ED discharge

Outcome	One-week risk	Four-week risk
Afraid of intimate partner	54.6%	66.3%
Verbal IPV	40.9%	59.4%
Physical IPV (hand, fists, feet)	4.6%	36.4%
Weapon-related IPV	9.1%	24.8%
Sought medical care for IPV	31.8%	37.1%

IPV denotes intimate partner violence.

Details of the outcomes and risk calculations are provided in the Methods section.

Analyses stratified according to the recentness of IPV at baseline indicated that the risk of sustaining physical abuse within four weeks of discharge was not considerably different among subjects who had presented for an IPV-related injury or illness (31.4%) versus subjects whose presenting condition was not due to IPV (38.5%). Similarly, the risk of seeking medical care for a condition stemming from IPV sustained within four weeks of discharge was not considerably different among subjects who had presented at baseline for an IPV-related injury or illness (42.9%) versus subjects whose presenting condition was not due to IPV (34.0%).

## Conclusion

This study found that approximately three of every four subjects participated during the follow-up phase of the study, and approximately two of every four subjects completed all follow-up reports to the automated telephone survey as was requested. This level of participation is encouraging given that patients in abusive relationships pose considerable challenges to follow prospectively as research subjects after hospital discharge. Although a key challenge from a methodologic perspective is to minimize attrition during follow up, the primary challenge pertains to subject safety and the fact that participation is believed to put subjects at risk for IPV recurrence. We chose an automated telephone survey system as a method of follow up because it has features that helped to minimize both attrition and risks to subjects. The subjects themselves chose when to engage the survey, and could report to the survey at any time of day or night as was convenient. Also, subjects could report from any touchtone telephone, and entered their responses by pressing numbers on the telephone keypad rather than by speaking out loud. Compared to cohort studies that have used investigator-initiated phone calls to subjects[5] or reviews of hospital records[4] as methods of identifying instances of IPV recurrence during follow-up, the features of an automated telephone survey obviate the need for the researcher to initiate contact with the subject and also enable detection of types of IPV that do not result in a hospital visit. Thus, the automated telephone survey confers advantages in terms of patient safety and data specificity and validity.

It is important to emphasize our impression that the incentive delivery method had much to do with achieving the good subject retention rate. By wirelessly recharging, onto the same convenience store debit card that the subject was given during the interview in the ED, additional disbursements shortly after each report to the automated telephone survey, the incentive came to be in the subject's possession with no exchange of a physical commodity from the investigator to the subject (e.g., by mailing a gift card, or asking the subject to pick a gift card up from the investigator). Our primary reason for establishing this delivery method was to minimize the likelihood of revealing to the subject's partner or anyone else the subject's participation in the study, which could put the subject at risk to be abused. But we expect that this delivery method had the additional benefit of being appreciated by the subject simply for its convenience, and thus this may have fostered participation during the follow-up phase. It is interesting to consider that varying the amount of the incentive depending upon how many reports have been made, and in particular offering a bonus for complete participation, may influence participation. The finding that many subjects made all four reports to the survey as requested, but few subjects made a total of two or three calls, provides evidence to this effect. In sum, based on experiences here, our perspective is that the good subject retention rate should be attributed to use of the automated telephone survey in conjunction with the specific incentive delivery method. We would encourage further investigation of this matter, however, perhaps through randomized trials to compare different incentive amounts and protocols to determine the role of the incentive in subject retention.

We reported that all subjects granted the investigator permission to call the subject by telephone during the follow-up period, as a way to remind the subject of the telephone number for the automated telephone survey in case the information card containing that number had been lost. Despite their willingness and despite finding no evidence that the few subjects who the investigator did call back were put at risk in doing so, it is this type of investigator-initiated contact that we are hoping to avoid. To be prudent, investigators should also consider the potential that a subject may give permission to be called out of a willingness to aid the research process, which may be heightened if the subject feels vulnerable and/or perceives the investigator or their involvement in the study as something that provides hope; and that in giving permission the subject may, consciously or not, put oneself at some degree of risk of detection by their partner as a research participant. The only way to eliminate the potential for this eventuality is to refrain from initiating contact.

The automated telephone survey technology employed here – IVR – has been used to study a range of health issues[14] and has been compared to other methods, including follow-back and daily diary methods, to assess the validity of data [15-17]. To our knowledge, however, this is the only prospective follow-up study of ED patients that used an automated telephone survey to assess for recurrence of IPV and medical care seeking behavior during the days and weeks following discharge. There is evidence that IVR fosters honest reporting more than does a telephone survey conducted by a live interviewer [18], and so it is possible that this may be an additional advantage of the follow-up method described here relative to the follow-up method of having the investigator make phone calls to subjects. As an alternative to IVR, subjects could be asked to report via computer to an Internet-based survey. The feasibility of this alternative may vary across communities depending upon the availability of computers and Internet access, and is worthy of being evaluated.

This study has limitations. It is possible that subjects who sustained abuse during follow-up were more inclined than others to make reports to the phone survey; also, subjects who did not sustain abuse during follow-up may have been less inclined to make reports. We considered contacting all subjects once the follow-up period had concluded, to ask for their impressions of whether their participation was related to their feelings of safety and their experiences with abuse during the follow-up period. We chose not to do so in order to avoid the chance for such contact to reveal the subject's participation to their partner. As a result, however, it is unclear how these sources of potential misclassification bias could affect the results of studies that in the future may use such a protocol. Also, the observed recurrence data suggest that ED patients in abusive relationships may face significant risks of sustaining recurrent abuse and of requiring medical care. Further, there is evidence that among patients who are in abusive relationships, the recurrence risk may be high regardless of how recently they were last abused and regardless of whether their chief complaint is IPV-related. If this is the case, IPV protocols should involve not only universal screening but ED exit planning conducted with each identified patient, regardless of IPV recency. However, this was a feasibility study based on a small number of subjects and was not intended to establish the incidence of these recurrence outcomes. Also, bias from differential participation (i.e., at baseline) and differential response (i.e., during follow-up) are threats to the validity of the study findings. We do not report the results of comparisons between participants and non-participants, or between responding and non-responding participants, because of small sample size and very limited power. For the same reason we do not statistically test for differences between those presenting to the ED with IPV versus those presenting for another reason. In the event of differential responding in future, large studies, informative dropout statistical methods may help alleviate this as a source of bias [19]. Also, the IVR survey was hosted for a cost of (U.S.) $2,500. The majority of this fee was allocated to the recording of the survey script by a professional voice talent and to the programming of the IVR system; the costs for hosting the system were minimal, and with the system in place, the amount required to manage responses from additional subjects to complete a larger study would have been nominal. With Internet-based survey options that are free, however, these should be evaluated for their feasibility as alternatives to IVR, or for use in conjunction with an IVR survey. Finally, with a follow-up period limited to four weeks, this study does not indicate whether subjects would have continued to make reports to the IVR system for longer periods of time. If investigators are interested in following subjects in the context of IPV for longer periods, additional feasibility research is warranted.

We conclude that an automated telephone survey holds promise as a means of learning about patient victims of IPV after they leave the ED, and that this method is warranted for use in population-based studies of IPV. The protocol tested during this study, specifically the use of an automated telephone survey in conjunction with a wireless incentive delivery system, enabled overcoming many practical and ethical obstacles [3] faced when considering how to study IPV in a patient population and appears to provide a feasible way of obtaining information from patient subjects after discharge. Applications of this method could aim to establish population-based estimates of the incidence of abuse after discharge, learn about steps patients take after discharge to minimize risks for abuse (e.g., leave home; go to a shelter), and test the efficacy of ED-based interventions aimed at improving health outcomes and preventing recurrence among victims of abuse [2]. This type of information would considerably advance knowledge in this field of research and would address the need to improve the ED-based response to IPV [3,6]. The protocol applied here could be equally beneficial for the study of other injury or disease types, especially when it is critical to monitor sensitive outcomes discretely.

## Abbreviations

ED: Emergency Department;

IPV: Intimate partner violence;

IVR: Interactive voice response;

SD: Standard deviation.

## Competing interests

The author(s) declare that they have no competing interests.

## Authors' contributions

DW, TR, and ME conceived of the study. ED, BC and DW designed the clinical protocol and DW, TR and ME designed the follow-up protocol. DW conducted patient interviewing and managed and analyzed the data. ME provided statistical advice. DW drafted the manuscript and all authors contributed to its revision. DW obtained the research funding and is responsible for the paper overall.
